# Aspirin Treatment of Mice Infected with *Trypanosoma cruzi* and Implications for the Pathogenesis of Chagas Disease

**DOI:** 10.1371/journal.pone.0016959

**Published:** 2011-02-15

**Authors:** Shankar Mukherjee, Fabiana S. Machado, Huang Huang, Helieh S. Oz, Linda A. Jelicks, Cibele M. Prado, Wade Koba, Eugene J. Fine, Dazhi Zhao, Stephen M. Factor, J. Elias Collado, Louis M. Weiss, Herbert B. Tanowitz, Anthony W. Ashton

**Affiliations:** 1 Division of Parasitology, Department of Pathology, Albert Einstein College of Medicine, New York City, New York, United States of America; 2 Department of Biochemistry and Immunology, Institute of Biological Sciences, Federal University of Minas Gerais, Belo Horizonte, Brazil; 3 Center for Oral Health Research, University of Kentucky Medical Center, Lexington, Kentucky, United States of America; 4 Department of Nuclear Medicine and the M. Donald Blaufox Laboratory for Molecular Imaging, Physiology and Biophysics, Albert Einstein College of Medicine, New York City, New York, United States of America; 5 Division of Infectious Disease, Department of Medicine, Albert Einstein College of Medicine, New York City, New York, United States of America; 6 Department of Pathology, University of São Paulo, Ribeirão Preto, Brazil; 7 Division of Perinatal Research, Kolling Institute for Medical Research, University of Sydney, Sydney, Australia; New York University School of Medicine, United States of America

## Abstract

Chagas disease, caused by infection with *Trypanosoma cruzi*, is an important cause of cardiovascular disease. It is increasingly clear that parasite-derived prostaglandins potently modulate host response and disease progression. Here, we report that treatment of experimental *T. cruzi* infection (Brazil strain) beginning 5 days post infection (dpi) with aspirin (ASA) increased mortality (2-fold) and parasitemia (12-fold). However, there were no differences regarding histopathology or cardiac structure or function. Delayed treatment with ASA (20 mg/kg) beginning 60 dpi did not increase parasitemia or mortality but improved ejection fraction. ASA treatment diminished the profile of parasite- and host-derived circulating prostaglandins in infected mice. To distinguish the effects of ASA on the parasite and host bio-synthetic pathways we infected cyclooxygenase-1 (COX-1) null mice with the Brazil-strain of *T. cruzi*. Infected COX-1 null mice displayed a reduction in circulating levels of thromboxane (TX)A_2_ and prostaglandin (PG)F_2α_. Parasitemia was increased in COX-1 null mice compared with parasitemia and mortality in ASA-treated infected mice indicating the effects of ASA on mortality potentially had little to do with inhibition of prostaglandin metabolism. Expression of SOCS-2 was enhanced, and TRAF6 and TNFα reduced, in the spleens of infected ASA-treated mice. Ablation of the initial innate response to infection may cause the increased mortality in ASA-treated mice as the host likely succumbs more quickly without the initiation of the “cytokine storm” during acute infection. We conclude that ASA, through both COX inhibition and other “off-target” effects, modulates the progression of acute and chronic Chagas disease. Thus, eicosanoids present during acute infection may act as immunomodulators aiding the transition to and maintenance of the chronic phase of the disease. A deeper understanding of the mechanism of ASA action may provide clues to the differences between host response in the acute and chronic *T. cruzi* infection.

## Introduction

In Latin America millions of people are at risk of infection with the parasite *Trypanosoma cruzi*, the cause of Chagas disease. The cardiac manifestations are the most prominent symptoms of disease. Acute myocarditis is accompanied by an intense inflammatory response including upregulation of inflammatory mediators such as cytokines, chemokines, nitric oxide and endothelin-1 [1]–[6]. As the acute infection wanes individuals may remain asymptomatic; however, 10 to 30% of infected individuals ultimately develop chronic cardiomyopathy [7]. Manifestations during this stage of the disease include congestive heart failure, conduction abnormalities and thrombo-embolic events [7], [8]. The etiology of the chronic cardiomyopathy is primarily the result of parasite persistence but may also result from microvascular spasm with focal ischemia and autoimmune mechanisms [4], [5], [9]–[11]. Our group has investigated the etiology of vascular spasm in the setting of *T. cruzi* infection. In this regard, we suggested as early as 1990 that the eicosanoid, thromboxane (TX)A_2_ contributed to *T. cruzi*-associated vasospasm and platelet aggregation [12].

Eicosanoids are a family of lipid mediators that participate in a wide range of biological activities including vascular tone, inflammation, ischemia and tissue homeostasis [13]. In mammals, the biosynthetic pathways for these important biological mediators are well described. Arachidonic acid (AA), derived from membrane phospholipids on the inner leaflet of the plasma membrane by phospholipase A_2_, is hydrolyzed by the prostaglandin (PG)H synthase/cyclooxygenase (COX) enzymes to PGH_2_ [14]. PGH_2_ is the central substrate for subsequent eicosanoid synthesis which is mediated by species-specific synthases to generate PGs and TXA_2_ [15]. Enzymes in the COX family are structurally and enzymatically similar but have different pathophysiological roles. COX-1 is constitutive and mediates gastric mucus production, platelet activation and vascular tone while COX-2 is inducible and functions in inflammation, cancer and tissue damage [13], [14]. The relevance of these enzymes, and the bioactive lipids they produce, are not well understood in parasitic disease.

Phospholipase A1 (PLA1) the enzyme that initiates the AA metabolic pathway by cleaving the Sn-1 acyl chain was reported in *T. brucei* [16], [17]. PGF_2α_ synthases have been identified in *Leishmania*, *T. cruzi* (Old Yellow Enzyme) and *T. brucei* [18], [19]. PGF_2α_ is the predominant eicosanoid species produced in *Leishmania* and *T. brucei*, along with smaller quantities of PGE_2_ and PGD_2_ [16], [18], [20]. Importantly, *T. cruzi* preferentially synthesizes TXA_2_ [21]. Eicosanoids released by *T. cruzi* may contribute to parasite differentiation, phagocytosis [22] and host survival [23] by acting as immunomodulators to aid transition and maintenance of the chronic phase of the disease. Moreover, recent studies have demonstrated that trypanosomes are capable of AA metabolism complicating the interpretation of the potential significance and source of these bioactive lipids [18]–[20].

Our recent data [21] indicated that host- and parasite-derived prostaglandins potentially contribute to the pathogenesis of Chagas disease. Given the increasing importance of eicosanoids in *T. cruzi* infection, it is not unexpected that there should be interest in non-steroidal anti-inflammatory agents (NSAIDS) in the pathogenesis and clinical management of this infection. However, administration of NSAIDS may enhance mortality in patients [23], [24] and in experimental *T. cruzi* infection [25]. Moreover, characterization of COX inhibition on disease exacerbation in *T. cruzi* has not been fully addressed. We sought to determine what effect NSAID use would have on the development of acute and chronic Chagas disease. To examine the consequences of COX inhibition we administered aspirin (ASA) to *T. cruzi* infected mice either early in the course of disease, 5 days post infection (dpi) or late in infection (60 dpi). ASA exhibits irreversible inhibition of COX isoforms and is widely used to treat the symptoms of Chagas disease making it the most clinically applicable choice for these studies. COX inhibition early in the disease increased parasitemia and mortality. Administration of ASA during the chronic phase had no effect on mortality or parasitemia but improved ejection fraction. ASA ablated the increased release of PGF_2α_ and TXA_2_ in response to *T. cruzi* infection; however, infection of COX-1 null mice only mimicked the effects of ASA on parasitemia, primarily through decreased TXA_2_ release. The enhanced mortality in response to ASA was likely due to “off-target” effects of ASA. ASA treatment of *T. cruzi* infected mice suppressed TNF-α release through increased expression of suppressor of cytokine signalling-2 (SOCS-2) and reduced TNF-α receptor-associated factor (TRAF6) expression in the spleen. Thus, the effects of ASA in *T. cruzi* infection may be via dual mechanisms that operate during different phases of disease.

## Results

### Global inhibition of eicosanoid production early in the course of *T. cruzi* infection results in increased parasitemia and mortality

**Figure 1:**
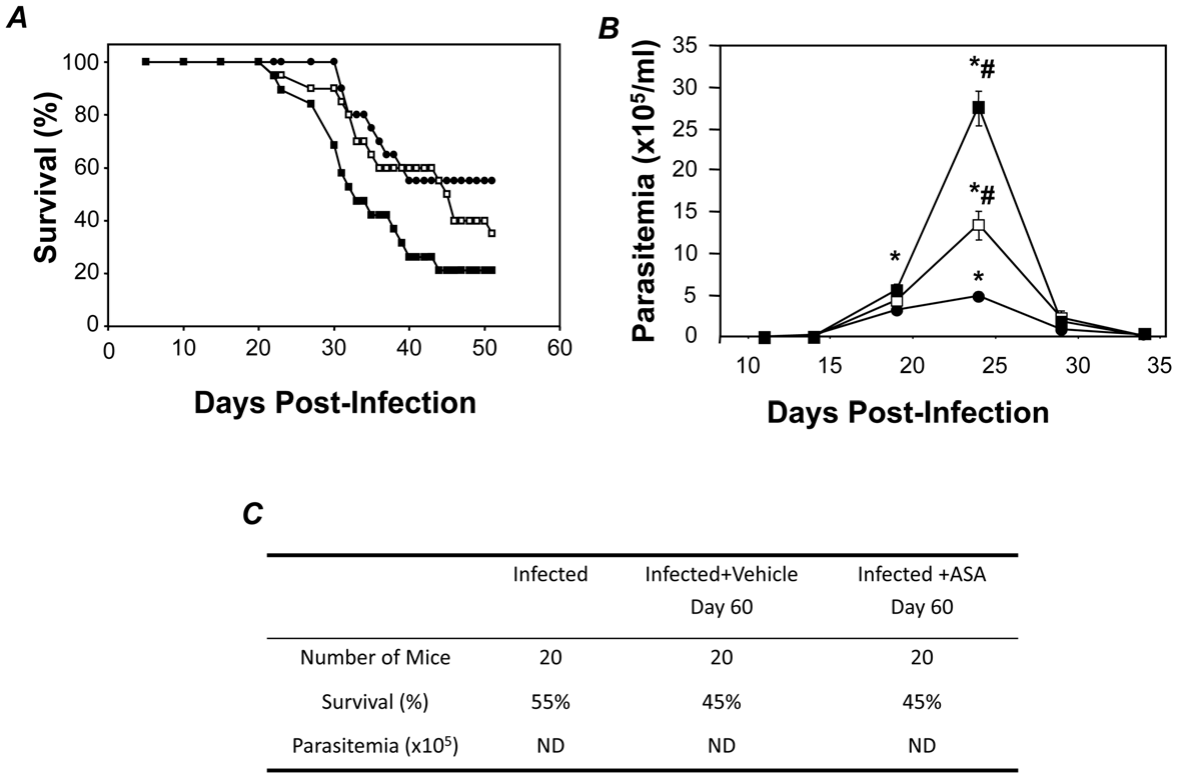
Early administration of ASA increases mortality and parasitemia in response to *T. cruzi* infection. ***A and B*** *.* CD-1 mice were infected with the Brazil strain of *T. cruzi* and mortality (***A***) and parasitemia (***B***) assessed in vehicle (•) and ASA treated mice (□, 20 mg/kg; ▪, 50 mg/kg) over 55 days post infection (dpi). Treatment with ASA or vehicle started at 5 dpi. ***C***. Table showing the effects of delayed ASA treatment on chronic experimental *T. cruzi* infection. ASA treatment (20 mg/kg) was initiated 60 dpi until 120 dpi. Survival and parasitemia were assessed 120 and 75 dpi respectively. Data are represented as mean ± SD are representative of at least 20 mice per group. * and # indicates significance (*P*≤0.05) from uninfected and infected mice, respectively. ND = not detected.

Infected CD-1 mice were treated with either 20 or 50 mg/kg ASA from 5 dpi to address the involvement of COX-derived mediators during acute infection. Over the subsequent 50 days of infection 40% of untreated mice died (Figure 1A). ASA treatment increased mortality during acute infection in a dose dependent manner with 60% and 80% mortality (50 dpi) in the groups treated with 20 and 50 mg/kg ASA, respectively (Figure 1A). Similarly, ASA treatment increased the parasitemia during acute infection by 2.7 and 5.6 fold in the 20 and 50 mg/kg ASA treated groups, respectively (Figure 1B). Conversely, treatment of mice with ASA (20 mg/kg) during the chronic phase (60 dpi) produced no exacerbation of disease (Figure 1C). Delayed administration of ASA did not increase peripheral parasitemia nor did it augment mortality (Figure 1C). Thus, eicosanoid production during acute infection appears to modulate host response and disease evolution in favor of progression to the chronic state.

### ASA treatment during the chronic disease improves ejection fraction

**Figure 2:**
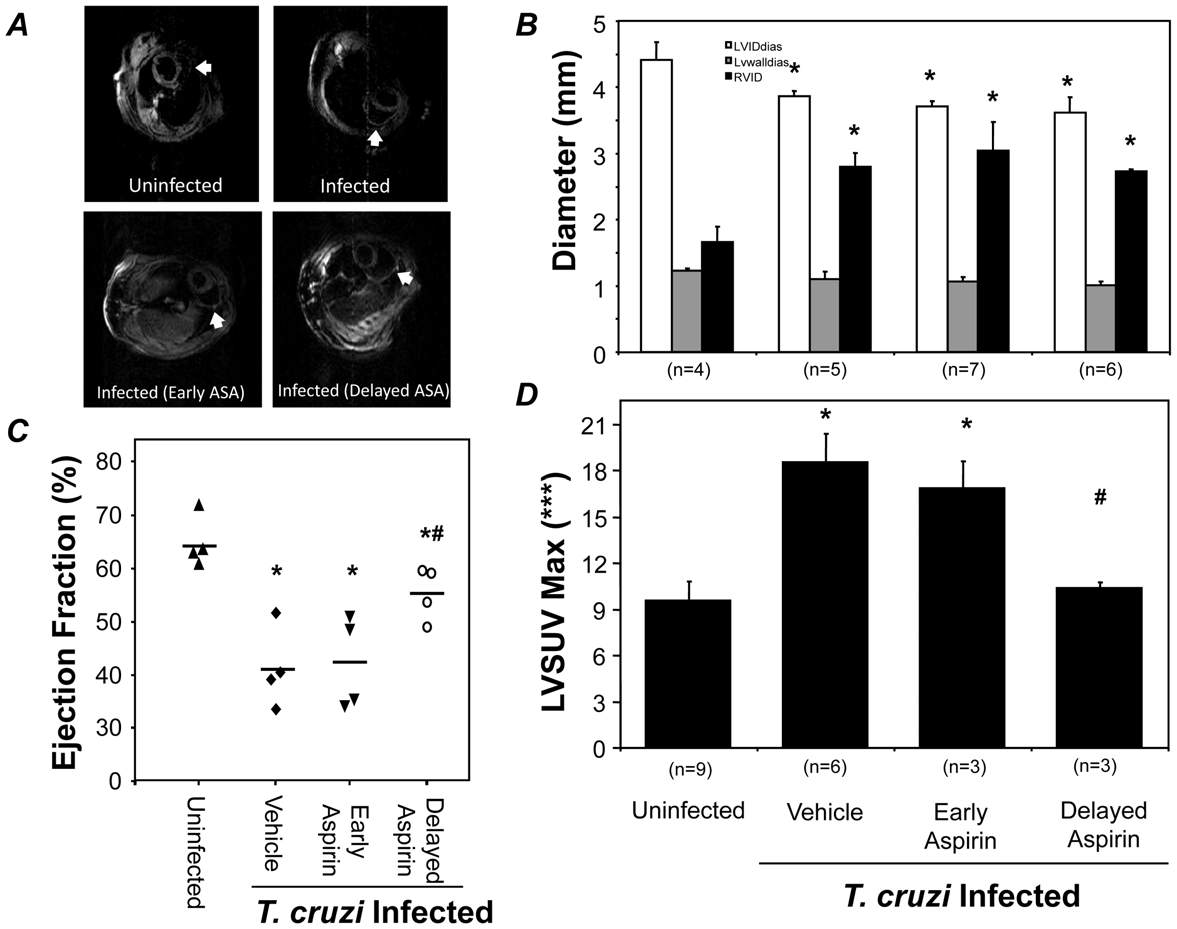
Effects of early and delayed administration of ASA on cardiac structure and function. ***A***. Short axis MRI images showing cardiac remodeling during *T. cruzi* infection of CD-1 mice with and without ASA treatment (20 mg/kg). Arrows indicate the wall of the right ventricle of the heart. ***B***. Cardiac dimensions were assessed in uninfected and infected with or without ASA at 5 (Early) or 60 (delayed) dpi. Parameters quantified included left ventricular internal diastolic diameter (***B***, while squares), left ventricular wall diameter (***B***, grey squares), right ventricular internal diameter (***B***, black squares). ***C***. Ejection fraction measured using echocardiography. ***D***. Left ventricular glucose SUV measured by microPET. Number of mice in each group is indicted. * and # indicates significance (*P*≤0.05) from uninfected and infected mice, respectively.

Declining cardiac function is a significant source of mortality in both experimental models and in patients suffering from Chagas disease. The effects of early (beginning at 5 dpi) or delayed (beginning at 60 dpi) treatment with ASA (20 mg/kg) on cardiac structure and function were determined using magnetic resonance imaging (MRI). Significant dilatation of the right ventricle was observed in mice in response to infection (Figure 2A). Neither early nor delayed treatment with ASA attenuated the right ventricular dilation (Figure 2A and B) with a 2 fold increase in internal diameter in all infected groups. Moreover, a 10% decrease in left ventricular diastolic diameter was also noted across all groups (Figure 2B). Despite this, delayed treatment significantly reversed the reduction in ejection fraction observed in infected mice as determined by echocardiography. The 38% decrease in ejection fraction in infected mice was attenuated to 15% upon delayed treatment with ASA. The improved ejection fraction in the delayed treatment group may be due to reduced inflammation during the chronic stage of the disease. Using microPET imaging we previously observed increased glucose uptake in the hearts of infected mice [26] which correlated with increased inflammation. Thus, our microPET data demonstrating reduced left ventricular glucose uptake (LVSUV; Figure 2D) in the delayed treatment group suggest reduced inflammation in that group compared with untreated infected mice. Unlike the mortality data, treatment at the early timepoints did not restore cardiac function indicating that the therapeutic window for the preservation of cardiac function and host survival are not the same.

**Figure 3:**
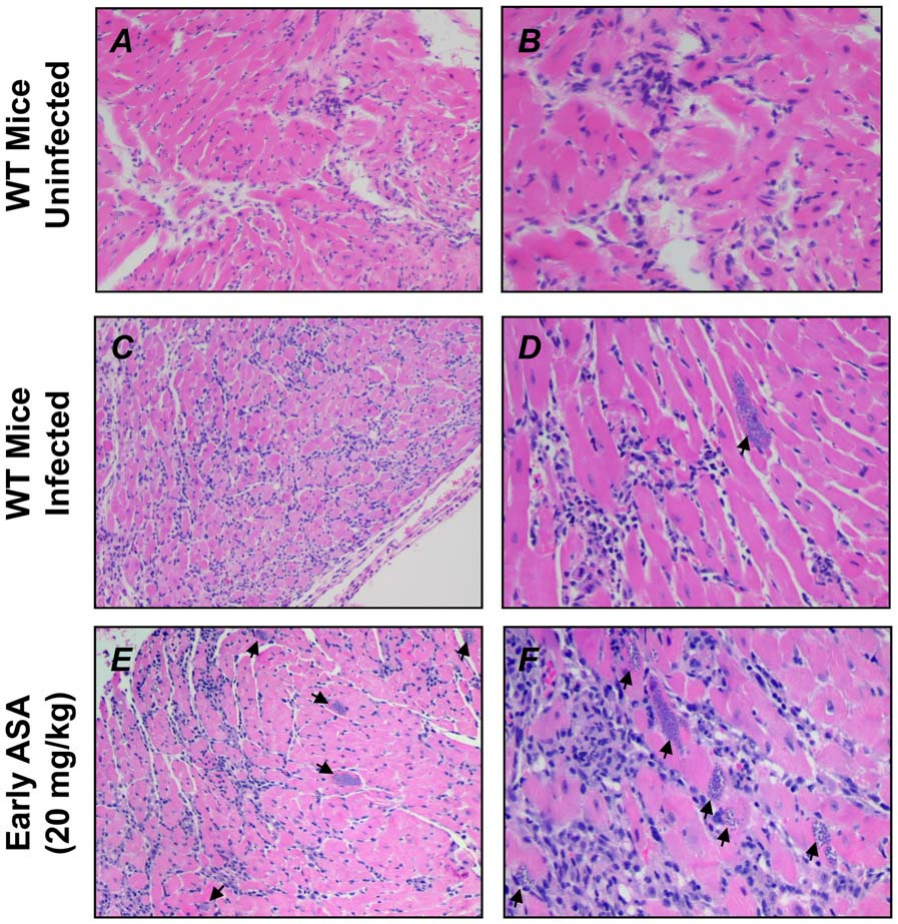
Cardiac pathology in ASA-treated mice is no different to vehicle treated control*s*. Representative histopathology of infected CD-1 mice with and without ASA treatment (20 mg/kg) at 35 dpi compared with uninfected controls. Sections were stained with H&E. Parasite pseudocysts are observed (arrows). Total magnification of either 100× (***A, C, E***) or 400× (***B, D, F***). Images are representative of al least five mice in each group.

The effective preservation of myocardial function by ASA prompted us to examine cardiac tissue from infected mice for hallmarks of disease (Figure 3). Compared to hearts from uninfected mice (Figure 3A and B) infected mice displayed increased inflammation and pseudocysts along with increased cellularity, mostly resulting from influx of inflammatory cells (Figure 3C and D). No significant differences in myocardial histopathology were observed between infected mice with or without ASA treatment with parasite pseudocysts present in both groups (arrows, Figure 3C through F). Thus, the action of ASA did not prevent the pathological changes to the heart induced by experimental *T. cruzi* infection.

### ASA treatment ablates eicosanoid production by both the parasite and host

**Figure 4:**
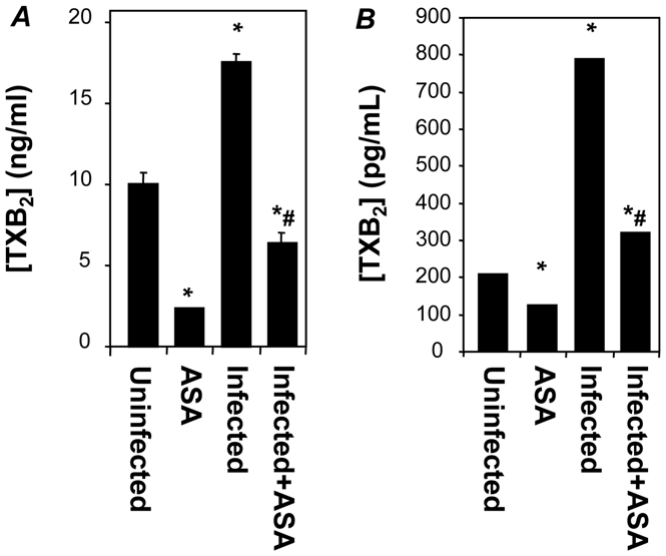
ASA inhibits both host- and *T. cruzi*-derived prostaglandin production. Plasma TXA_2_ levels, measured as the stable hydrolytic product TXB_2_ by ELISA, in uninfected or infected CD1 (***A***) or TXA_2_ synthase null (***B***) mice. ASA treatment (20 mg/ml) was initiated on 5 dpi. Circulating levels were assessed at 30 and 20 dpi, respectively. Data (mean ± SD) are derived from at least 5 mice per group. * and # indicate significance (*P*≤0.05) from uninfected mice and infected mice, respectively.

The improvements to cardiac function with ASA without alteration to cardiac pathology or structure indicated a humoral mediator in the suppression of cardiac function. TXA_2_ is an ASA sensitive mediator with robust links to cardiac damage post-infarction and during failure [27], [28]. To determine the extent of COX suppression by ASA treatment we measured TXA_2_ levels (as the stable hydrolytic product TXB_2_) in the plasma of infected mice by ELISA. TXB_2_ levels in infected CD-1 mice increased linearly from 10 dpi and peaked at 27 ng/ml plasma at 45 dpi (Figure 4A). In ASA-treated, infected mice TXA_2_ levels remained at or below those observed in uninfected mice (constant at 8 ng/ml plasma over 45 dpi). ASA treatment ablated TXA_2_ release in uninfected mice (Figure 4A) and the augmentation of TXA_2_ release in ASA treated mice due to *T. cruzi* infection was also blunted when compared to vehicle treated controls. Thus, both host- and parasite-derived eicosanoid synthesis in infected mice appear to be sensitive to COX-inhibition by ASA.

Previously we demonstrated that in *T. cruzi*-infected TXA_2_ synthase null mice the majority of TXB_2_ in the plasma is derived from the parasite, and not the host [21]. TXA_2_ release in infected TXA_2_ synthase null mice (Figure 4B) was also ablated by treatment with ASA (20 mg/kg). The reduction in total TXA_2_ levels indicated that either the TXA_2_ biosynthetic pathway in the parasite is significantly inhibited by ASA or that the parasite is dependent upon host-cell derived PGH_2_. Treatment of cultured epimastigotes, the extracellular life-stage of *T. cruzi*, with ASA had no effect on parasite proliferation *in vitro* (data not shown). These results were consistent with previous data suggesting that the biosynthetic pathways of the parasite are resistant to the effects of ASA [18], [20], [29]. Thus, it appears that the scavenging of prostanoid precursors by the parasite from the host was the most likely hypothesis for the observed effects.

### Infection of COX-1 and TXA_2_ Synthase null mice mimics only some of the changes in ASA treated mice

**Figure 5:**
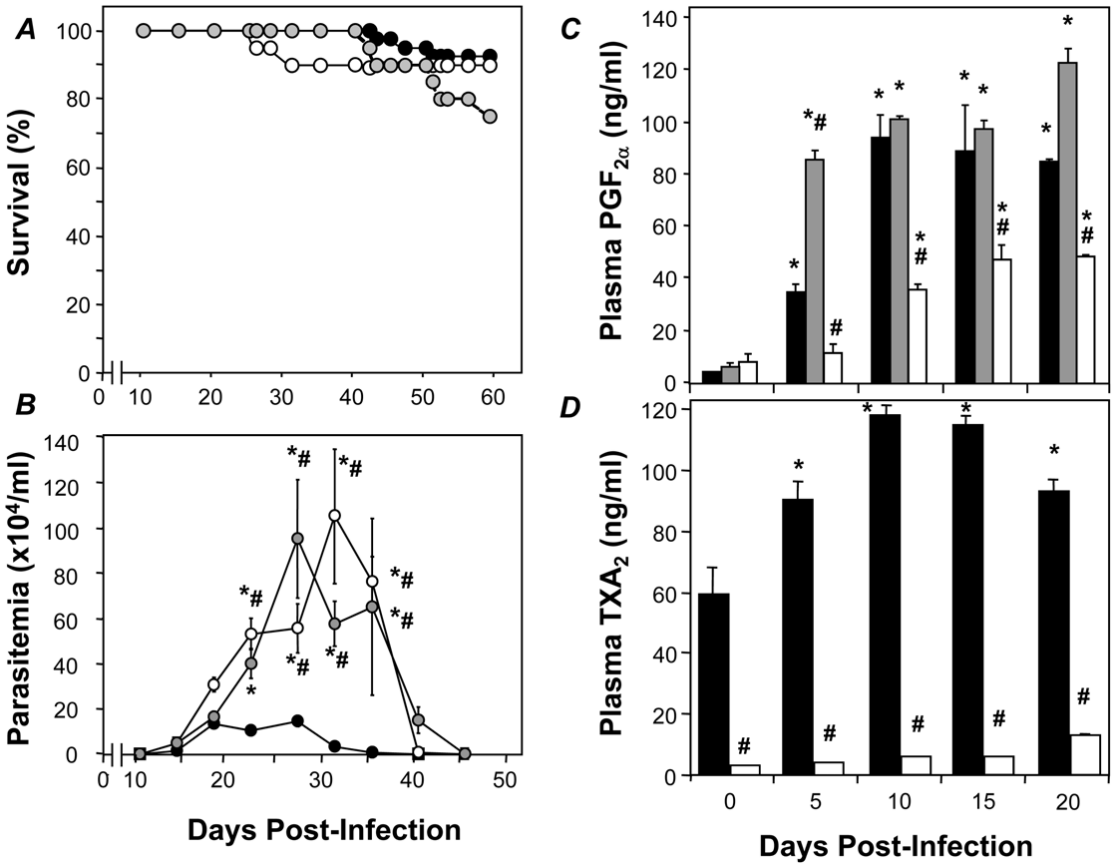
Deletion of COX-1 mimics the effects of ASA on parasitemia but not survival in *T. cruzi* infected mice. ***A*** and ***B*** *.* Survival curves (***A***) and peripheral parasitemia (***B***) for WT (black circle), TXA_2_ synthase null mice (grey circle) and COX1 null mice (○) mice after inoculation with 10^5^ trypomastigotes of the Brazil strain. ***C*** and ***D*** *.* Measurement of plasma PGF_2α_ (***C***) and TXB_2_ (***D***) in infected WT (black square), TXA_2_ synthase null mice (grey square) and COX-1 null mice (white square) mice. Data are represented as mean ± SD are representative of at least 20 mice per group. * and # indicates significance (*P*≤0.05) from uninfected and infected WT mice, respectively.

To confirm the mechanism of ASA action *in vivo* we examined the pathogenesis in genetically modified mice with attenuated biosynthetic capacity. ASA has a 66 fold preference for COX-1 over COX-2 [30], [31]; therefore, COX-1 null mice were chosen to test the hypothesis. TXA_2_ synthase null mice (normal COX-1 activity by ablated TXA_2_ synthesis) were used for comparison. Infection of COX-1 and TXA_2_ synthase null mice with *T. cruzi* yielded small alterations in the mortality curves but no significant change in the overall survival compared to wild-type (WT) littermates (Figure 5A). Conversely, peripheral parasitemia in infected COX-1 null mice was increased 9.2 fold compared to WT littermates with peak parasitemia increased 5.7 fold and prolongation of parasite levels from 33 dpi to 42 dpi. Similarly, parasitemia in *T. cruzi* infected TXA_2_ synthase null mice was increased 7.1 fold over the 50 dpi and accompanied by a 7- fold increase in peak parasitemia and prolongation of parasite persistence until 48 dpi. The kinetics in both genetically modified mouse strains were almost identical suggesting that the majority of these changes were accounted for by a lack of TXA_2_ production by the host in COX-1 null mice.

Eicosanoid release from infected COX-1 and TXA_2_ synthase null mice was significantly different from their WT controls. Plasma of infected COX-1 null mice exhibited a 2.1 fold decreased rate of synthesis and an overall 70% decrease in PGF_2α_ production (Figure 5C) compared to infected WT mice. Unlike WT mice, the plasma of COX-1 null mice did not contain significant levels of TXA_2_ (Figure 5D). As stated above, our previous data suggested that most of the circulating TXA_2_ in experimental infection is parasite derived [21]. Thus, our current data suggest that scavenging of metabolic intermediates from the host is likely required for parasite eicosanoid biosynthesis. To further support this hypothesis the release of PGF_2α_ from infected TXA_2_ synthase null mice, which have unaffected generation of precursor molecules, was normal (Figure 5C). Collectively, these data indicate that decreased eicosanoid biosynthesis by ASA only accounts for the control of parasite proliferation in experimental infection while the enhanced mortality observed with early administration of ASA may be a response to alterations in other unrelated pathways.

### Effect of ASA on cytokines in *T. cruzi* infected mice

**Figure 6:**
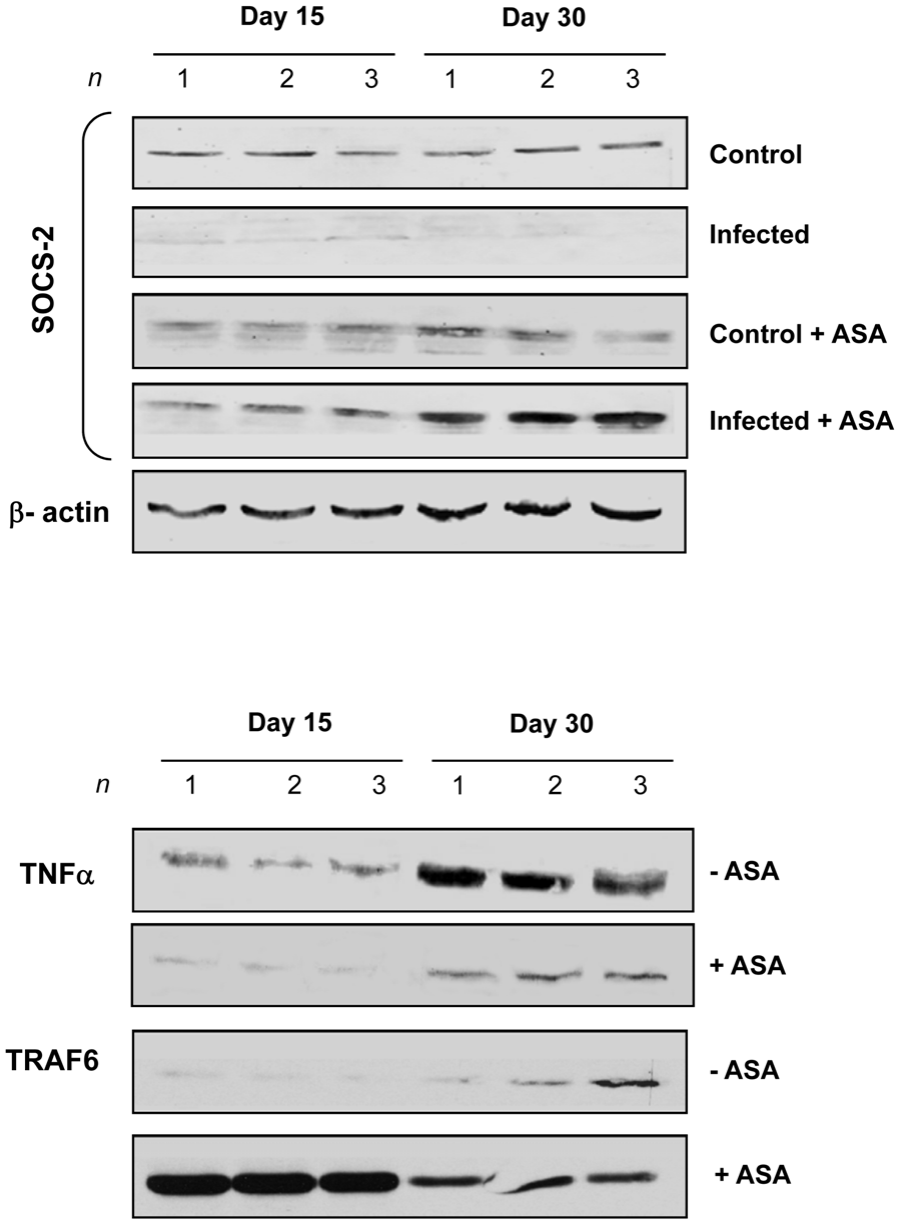
Treatment of *T. cruzi-*infected mice with ASA modulates cytokine signaling in the spleen of infected mice. Splenic extracts were prepared from uninfected and infected CD1 mice treated with ASA (20 mg/kg) from 5 dpi. Immunoblotting for TNF-α, SOCS-2 and TRAF-6 was performed on 15 and 30 dpi. β-actin was used as a loading control. Immunoblots are representative from at least three separate experiments.

The enhanced mortality when ASA treatment was initiated early resulted in the hypothesis that ASA treatment might inhibit priming of the innate immune system in order to decrease host response to the parasite. Plasma of mice treated with ASA (20 mg/kg) during acute *T. cruzi* infection displayed a reduction in TNF-α and IFN-γ levels 15 dpi (data not shown). Immunoblotting of lysates from the spleen of infected mice confirmed that TNF-α expression was significantly reduced by ASA treatment (Figure 6). These findings were not replicated in cardiac tissue (no change in TNFα production compared to uninfected mice) indicating that the spleen was the likely source of the cytokines present in the plasma and the target organ of ASA treatment. ASA has many off-target effects that influence the inflammatory response including inhibition of NFκB [32], augmentation of SOCS-2 and decrease of TRAF6 expression [33], [34]. Treatment with ASA early in infection not only ablated TNF-α release from spleen but also increased SOCS-2 and reduced TRAF6 expression (Figure 6). Changes were observed as early as 15 dpi and were even more pronounced at 30 dpi indicating they were present throughout the acute phase of disease. Thus, the enhanced mortality observed in infected mice treated with ASA early in infection may be due to suppression of the innate immune response as a result of suppression of cytokine synthesis/release and receptor signaling associated with the initial “cytokine storm” during acute infection.

**Figure 7:**
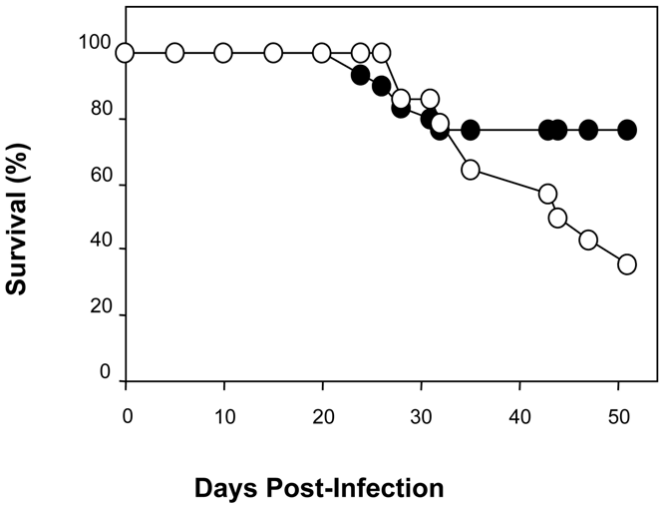
Treatment of *T. cruzi* infected COX-1 null mice with ASA increases mortality. Survival curves for COX-1 null mice treated with vehicle (•; n = 10) or ASA (20 mg/kg) (○; n = 14) beginning on 5 dpi. Mice were inoculated with 10^4^ trypomastigotes of the Brazil strain and observed over 50 dpi.

To examine the above hypothesis more directly, we treated infected WT and COX-1 null mice with ASA (20 mg/kg) and examined the effects on survival (Figure 7). Infection of COX-1 null mice with *T. cruzi* had little effect on host survival with 23.3% mortality over the 50 days of infection. However, infected COX-1 null mice treated with ASA beginning 5 dpi significantly exacerbated mortality with 65% of mice dying in response to *T. cruzi* infection over the subsequent 50 days and with a linear rate of loss (1.995 mice/day) from day 26 onwards. As an important target of ASA was removed in these mice we can only speculate that the “off target” effects of ASA we have observed are those that mediate the mortality in response to infection.

## Discussion

It is now appreciated that the release of eicosanoids during infection with *T. cruzi* regulates host responses and controls disease progression [21]. The role of these bioactive lipids in acute and chronic Chagas disease is largely unexplored and potentially further complicated by whether the host or the parasite is the primary source of synthesis. In this study we found that ASA treatment increased mortality and parasitemia in a dose-dependent manner during acute infection with the Brazil-strain of *T. cruzi* in mice. These changes were due, in part, to suppression of eicosanoid production (primarily TXA_2_) which controls parasite proliferation and may participate in cytokine release/signaling during early disease. Importantly, our data strongly suggest that control of fever and pain with ASA during acute Chagas disease should be used with caution. Conversely, use of ASA in the chronic phase of disease may improve cardiac function suggesting the same COX-1 products that mediate host-survival during the acute disease are likely to contribute to the progression of cardiac damage and heart failure in the chronic phase. Importantly, we established an essential host-parasite interdependence that dictates the biosynthetic activity of the parasite. This interdependence is an exploitable target for therapy to manage the chronic phase of disease and potentially prevent disease progression.

Previous studies have attempted to document the role of eicosanoids in early disease using pharmacological intervention with mixed results [22], [24], [25], [35], [36]. Pharmacological antagonists selective for either COX-1 (ASA), COX-2 (celecoxib) or both (indomethacin) increase mortality and parasitemia (both peripheral blood counts and cardiac parasite nests) regardless of mouse or parasite strain used [23]–[25], [36]. Conversely, others have found inhibition of prostaglandin synthesis/release ablates parasitemia and extends survival in mice infected with *T. cruzi* [22], [35], [37]. Our data correlates well with the former group of studies regarding the changes in parasitemia and degree of mortality. Moreover, use of the COX-1 null mice in this study confirms that COX-1 derived mediators from the host contribute to the suppression of parasite proliferation but perhaps not mortality in acute disease. None of the other studies have utilized null mice to confirm the observed effects and therefore it is difficult to know whether mortality and parasitemia are coordinately regulated in other reports or the response to separate properties of the pharmacological antagonists used (as suggested by our study).

The mechanism for the enhanced mortality with NSAID treatment during acute disease may lie with more complete inhibition of prostaglandin synthesis or “off-target” effects of these agents. ASA is not mono-specific and will also inhibit COX-2 [38]. Conversely, the COX-1 null mice have “normal” COX-2 levels and synthesis of many of the most potent immunosuppressive prostaglandins, e.g. PGE_2_ and PGI_2_, are closely linked to COX-2 expression [35]. Therefore, a significant reason for why ASA, but not deletion of COX-1, might be lethal in mice is the presence of COX-2-associated immunosuppressive prostaglandins in the COX-1 null mice. Aside from the inhibition of prostaglandin synthesis ASA induces the synthesis of aspirin triggered lipoxin (ATL) which is COX-2-dependent with little contribution from COX-1. ALT induces SOCS-2 expression and TRAF6 degradation. Importantly, Machado and colleagues [34] demonstrated that ASA-treated SOCS-2 null mice given LPS by the intra-peritoneal route could not inhibit neutrophil migration and TNFα signaling. Thus, mortality may have more to do with modulation of the impending “cytokine storm” during acute disease than actual prostaglandin production.

The dichotomy over the effects of NSAIDS in acute disease might result from the different combination of agents, mice and parasite strains previously employed. The expression of both COX isoforms remains unchanged during infection and there is no increase in COX-2 levels in COX-1 null mice as detected by immunoblotting (data not shown). While the role of COX-2 in *T. cruzi* infection is largely undefined both COX-1 and -2 appear to play different roles during acute infection. Inhibition of COX-2 (celecoxib), but not COX-1(ASA), prevented the thrombocytopenia and leukopenia associated with acute infection and increased reticulocyte counts in response to infection [25]. Inhibition of COX-1 and -2 reciprocally regulates NO release from M1 and M2 macrophages which may correlate with resistance to disease. Consistent with this observation, COX-2-derived prostaglandins mediate most of the immunosuppressive effects during the initial phase of *T. cruzi* infection [35]. This may result from the observations that PGI_2_ and PGE_2_ are more closely linked to COX-2 metabolism while COX-1 is aligned with TXA_2_ synthesis [39], [40]. Thus, the selectivity of the NSAIDs used may determine whether parasite or host production of PGs is the primary target of the treatment regimen used.

Our data with COX-1 null mice and pharmacological antagonism strongly indicate that host-derived PGH_2_ is involved in PG synthesis throughout infection. A key question is whether the host or parasite is the primary source of the lipid mediators regulating the pathogenesis of disease. Pharmacological inhibition does not distinguish between these two sources of eicosanoids. The reduction in PGF_2α_ release in COX-1 null, but not TXA_2_ synthase null, mice indicates that COX activity in the host provides precursor molecules required for the biosynthetic pathways of this parasite. This “scavenging” hypothesis is confirmed by the inability of the parasite (the primary source of TXA_2_ during infection) to sustain TXA_2_ release in the COX-1 null mice. If the parasite is scavenging precursors from the host then they would only need the terminal synthases to produce bioactive lipids. Fatty acid biosynthetic pathways in trypanosomes are poorly defined and little homology is reported between the mammalian enzymes and their trypanosomal homologues [20]. Some putative candidates, such as the PGF_2α_ synthase “old yellow enzyme” [19], have been identified. However, reports have indicated that parasitic biosynthetic pathways are resistant to mammalian antagonists, such as ASA, which have little effect on parasite biology [18], [20], [29]. Conversely, the recent report of anti-parasitic activity of indomethacin derivatives [41] indicates that the active sites of parasite enzymes, if not their primary sequences, are sufficiently homologous to their mammalian counterparts. Recent structural characterization of the target enzyme (TcCYP51), which participates in sterol biosynthesis of *T. cruzi*, has facilitated understanding of the integral nature of this enzyme to *T. cruzi* and has revealed much of the kinetics of the mechanism of action of indomethacin amides [42]. Interestingly, no enzyme other than COX isoforms has been identified as sensitive to indomethacin. However, it remains to be determined whether TcCYP51 is an integral component of the eicosanoid biosynthetic pathway in *T. cruzi*. [41].

The identification of the PGH_2_ derivatives that are most important for disease remains unsolved. Several species of eicosanoids have been implicated in both acute and chronic Chagas disease. Plasma from infected mice displayed increased levels of PGF_2α_, PGI_2_, TXA_2_ [43] and PGE_2_ [36] compared to uninfected mice from 10 dpi onwards. Previously, we determined that the main prostaglandins derived from *T. cruzi* are TXA_2_ and PGF_2α_ [21], indicating that host is the likely source of the elevated PGI_2_ and PGE_2_. No specific role has been delineated for the elevated PGI_2_ and PGF_2α_ observed in plasma from experimental *T. cruzi* infection. PGF_2α_ levels in the TXA_2_ synthase null and WT mice were similar indicating this prostaglandin was likely not involved with the augmentation of parasitemia observed in the COX-1 null and ASA treated mice or in the regulation of mortality. This leaves the potential role of PGF_2α_ in Chagas disease largely unexplored; however, the significant amounts of PGF_2α_ produced by *T. cruzi*, and the fact that all members of the trypanosomatids have an identifiable synthase for PGF_2α_, indicate that it is of significant value to the parasite.

During acute infection, PGE_2_ has been shown to modulate the virulence of the *T. cruzi* strain. A non-lethal strain (K98) provoked elevated circulating PGE_2_ while lethal strains (RA or K98-2) did not [24]. Inhibition of COX activity (and therefore PGE_2_ release) increased mortality in K98-strain infected mice but PGE_2_ infusion did not attenuate the virulence of the RA strain. Inhibition of PGE_2_ synthesis reduces both inflammatory infiltrates and cardiac fibrosis during acute infection [37]. Conversely, preventing host response to parasite-derived TXA_2_ augmented death and parasitemia [21]. TXA_2_ likely regulates vasospasm, thrombosis, vascular permeability and endothelial cell dysfunction during acute disease. TXA_2_ also displays immunosuppressive properties as WT mice display minimal pathology but TXA_2_ receptor null mice exhibited pronounced myocardial inflammation with an almost 3-fold increased in parasite load in cardiac tissue. Thus, it appears that the eicosanoids present during acute infection largely act as immunomodulators that aid in the transition to and maintenance of the chronic phase of the disease [23]. It is unclear whether *T. cruzi* generates prostaglandins as a defense against host immune system or whether it hijacks the host prostaglandin metabolic pathway in its favor. To this end, further studies using null mice missing biosynthetic enzymes or receptors are required to fully elucidate the role of the identified prostaglandins in Chagas disease.

In contrast to acute infection, where plasma levels of multiple PGs are elevated, only increased levels of TXA_2_ are observed in chronic disease (>180 dpi) [43]. In chronic disease the effects of TXA_2_ largely promote tissue damage, especially in the heart where it may exacerbate myocyte apoptosis and enhance progression to dilated cardiomyopathy and heart failure, a major cause of death in patients with this disease. Thus, disproving the adage that the things that don't kill you make you stronger. In addition to the maelstrom of changes that TXA_2_ mediates during acute infection, the secretion of TXA_2_ would prevent the initiation of an adaptive immune response by the host [44], enabling progression to and maintenance of the chronic phase of the disease. Finally, the role for TXA_2_ in chronic disease is made more complicated by its control of parasite proliferation. While we have confirmed that TXA_2_ plays a prominent role in Chagas disease the hypothesis that parasite-derived TXA_2_ is the primary quorum sensor for the parasite [21] may need to be re-visited. Unlike in acute infection (<30 dpi) parasite-derived TXA_2_ release does not function to suppress peripheral parasitemia in the long term with overall parasite load in the TXA_2_ synthase null mice 7-fold higher than WT littermates. This produced a late increase in the mortality of TXA_2_ synthase null mice which was not significant but may point to a need for host-derived TXA_2_ for control of the severity of the chronic disease. In fact, in the group with delayed ASA treatment there was an improvement in the infection-associated decrease in ejection fraction which may have resulted from negating the detrimental effects of TXA_2_ on myocyte contractility, platelet function and vascular tone.

In conclusion, our results demonstrate for the first time, that parasitemia and mortality in response to COX blockade during *T. cruzi* infection may be due to different pathways related to inhibition of prostaglandin synthesis and cytokine release respectively. Our data also show, for the first time, an interdependence of the parasite on host metabolism for prostanoid biosynthesis. These findings advance our understanding of host–parasite relationships and reveal a potential new avenue for pharmacological treatment for a disease with few therapeutic options.

## Materials and Methods

### Mice

Male CD-1 mice were obtained from Charles River Laboratories (Wilmington, MA). C57Bl/6 and CH3/HeJ mice were obtained from Jackson Laboratories (Bar Harbor, ME). COX-1 null mice and their wild type counter parts (originally from Taconic Farms [Hudson, NY]), TXA_2_ synthase null (originally from Dr. Kenneth Wu, University of Texas Health Science Center, Houston, TX), were bred in our facility. This study was carried out in strict accordance with the recommendations in the Guide for the Care and Use of Laboratory Animals of the National Institutes of Health. All experiments involving mice were approved by the Albert Einstein College of Medicine Institutional Animal Care and Use Committee (Approval Number: 20100204). All efforts were made to minimize suffering during surgical procedures.

### Parasitology and patholog*y*

The Brazil strain of *T. cruzi* was used in our experiments. The Brazil strain was maintained in C3H/HeJ. Hearts were obtained from infected and uninfected mice, fixed in 10% buffered formalin, paraffin embedded and stained with H&E. Male mice (8–10 weeks) were infected by an intra-peritoneal route with 5×10^4^ trypomastigotes Brazil strain of *T. cruzi* at the inoculums indicated. ASA (Sigma-Aldrich, Saint Louis, MO) was administered daily via intraperitoneal route at a dose of either 20 or 50 mg/kg of body weight beginning 5 (early) or 60 (delayed) dpi and was continued for 60 days. Mortality was recorded and blood drawn for the determination of parasitemia at the intervals stated. Parasitemia was assessed by counting in a hemocytometer chamber.

### Cardiac magnetic resonance imaging (MRI)

Cardiac MRI of mice infected with *T. cruzi* was first described by our laboratory group [45]. Briefly, the mice were anesthetized with 1.5% of isoflurane. A set of standard, shielded, nonmagnetic electrocardiographic leads ending in silver wires were attached to the four limbs. The ECG signal was fed to a Gould ECG amplifier (Gould Instrument Systems, Inc. Valley View, OH) associated with the Ponemah Physiology data acquisition system (Gould Instrument Systems, Inc. Valley View, OH) for monitoring the ECG and the R wave triggered a 5 volt signal to gate the spectrometer. Images were acquired with a GE/Omega 9.4 T vertical wide-bore spectrometer operating at a 1H frequency of 400 MHz and equipped with 50-mm shielded gradients (General Electric, Fremont, CA) and a 40-mm ^1^H imaging coil (RF Sensors, New York, NY). Temperature within the coils was maintained at 30°C using a water cooling unit (Neslab Instrument, Inc., Portsmouth, NH). This temperature prevented hypothermia in the anesthetized mice. After attachment of the cardiac gating leads, the mice were wrapped in a Teflon sheet and multi-slice spin echo imaging was performed to obtain short axis images of the heart. The gating delay was adjusted to collect data in systole or diastole. The following parameters were used to obtain 8 short axis slices: echo time, 18 msec; field of view, 51.2 mm; number of averages, 4; slice thickness, 1 mm; repetition time, approximately 0.2 sec; matrix size, 128×256 (interpolated to 256×256).

Several sets of 8 slices were acquired to define the entire heart and to obtain images in diastole and systole taking approximately 20–30 minutes per mouse. Data were transferred to a PC and analyzed using MATLAB-based software. Left ventricle (LV) and right ventricle (RV) dimensions in millimeters were determined from the images representing end-diastole. The left ventricular wall is the average of the anterior, posterior, lateral, and septal walls. The right ventricular internal dimension is the widest point of the right ventricular cavity.

### Echocardiography

Echocardiography of mice infected with *T. cruzi* has been described previously by our laboratory [46]. Briefly, the mice were lightly anesthetized with 1.5% isoflurane in 100% O_2_; the chest wall was shaved and a small gel standoff was placed between the chest and a 30-MHz RMV-707 B scanhead interfaced with a Vevo 770 High-Resolution Imaging System (VisualSonics, Toronto, ON, Canada). High-resolution, two-dimensional electrocardiogram-based kilohertz visualization (EKV Mode) and B mode images were acquired. Continuous, standard electrocardiogram was recorded using electrodes placed on the animal's extremities. Diastolic measurements were performed at the point of greatest cavity dimension, and systolic measurements were made at the point of minimal cavity dimension, using the leading edge method of the American Society of Echocardiography (http://www.asefiles.org/ChamberQuantification.pdf). Ejection fraction was calculated and used as a determinant of LV cardiac function.

### Micro-positron emission tomography (microPET)

We were the first group to describe the utility of microPET in the mouse model of Chagas disease [26]. Briefly, the mice were imaged after 3 hours of fasting. Mice were anesthetized with 1.5% isoflurane-oxygen mixture, which continued throughout the imaging portion of the procedure. Each mouse was placed on a heating pad before and during scanning to maintain normal body temperature. Mice were administered 300–400 µCi (12–15 MBq) in 0.1 mL normal saline, [^18^F] fluoro-2-deoxyglucose (FDG), via tail vein and imaging was started at 1 hour after injection as previously described. Imaging was performed using a Concorde Microsystems R4 microPET Scanner, with 24 detector modules, without septa, providing 7.9 cm axial and 12 cm transaxial field of view. Acquisitions were performed in three-dimensional (3D) list mode. Image analysis was performed using ASIPRO VM (Concorde Microsystems, LLC) dedicated software. Manual regions of interest (ROI) were defined around areas of visually identified heart activity in the LV. Successive scrolling through 2 dimensional slices (each 1.2 mm thick in the axial images) permitted identification of a pixel of maximum measured decay-corrected uptake, termed the standardized uptake value, or SUV max. The SUV max is the maximum value of the percentage injected dose per gram of cardiac tissue multiplied by the body weight of each animal.

### Measurement of PGF_2α_ and TXB_2_ by ELISA

Blood was drawn from the retro orbital plexus in anti-coagulated tubes with heparin (100 U/ml). The blood was centrifuged at 1500xg to remove cellular components and the platelet-poor plasma used for the assessment of prostaglandins. Plasma TXA_2_ and PGF_2α_ levels were determined using sensitive ELISA kits according to manufacturer's instructions (Cayman Chemical Company, Ann Arbor, MI). TXA_2_ levels were determined by measuring the stable hydrolytic product TXB_2_.

### Immunoblotting of Signaling Molecule*s*

For immunoblotting, aliquots of whole cell lysates (30 µg) from spleen and heart were separated by SDS-PAGE under reducing conditions. Proteins were transferred onto nitrocellulose membrane (Protan BA 85 Nitrocellulose from Whatman, Dassel, Germany) and analyzed by immunoblotting using antibodies against SOCS-2, TNFα, and TRAF6 (Santa Cruz Biotechnology, Santa Cruz, CA). Antibodies against β-actin (Ana Spec Inc, San Jose, CA) was used to control for loading.

### Statistical Analysis

Data were pooled and statistical analysis was performed using the Mann-Whitney U-test using Sigma Stat Version 2.0. Statistical differences (p≤0.05) are indicated on each figure using * or # to denote significance from control and infected groups, respectively.
